# Cognitive behavioural treatment for women who have menopausal symptoms after breast cancer treatment (MENOS 1): a randomised controlled trial

**DOI:** 10.1016/S1470-2045(11)70364-3

**Published:** 2012-03

**Authors:** Eleanor Mann, Melanie J Smith, Jennifer Hellier, Janet A Balabanovic, Hisham Hamed, Elizabeth A Grunfeld, Myra S Hunter

**Affiliations:** a Institute of Psychiatry, King's College London, UK; b Guy's and St Thomas' NHS Foundation Trust, London, UK; c School of Psychology, University of Birmingham, Birmingham, UK

## Abstract

**Background:**

Hot flushes and night sweats (HFNS) affect 65–85% of women after breast cancer treatment; they are distressing, causing sleep problems and decreased quality of life. Hormone replacement therapy is often either undesirable or contraindicated. Safe, effective non-hormonal treatments are needed. We investigated whether cognitive behavioural therapy (CBT) can help breast cancer survivors to effectively manage HFNS.

**Methods:**

In this randomised controlled trial, we recruited women from breast clinics in London, UK, who had problematic HFNS (minimum ten problematic episodes a week) after breast-cancer treatment. Participants were randomly allocated to receive either usual care or usual care plus group CBT (1:1). Randomisation was done in blocks of 12–20 participants, stratifying by age (younger than 50 years, 50 years or older), and was done with a computer-generated sequence. The trial statistician and researchers collecting outcome measures were masked to group allocation. Group CBT comprised one 90 min session a week for 6 weeks, and included psycho-education, paced breathing, and cognitive and behavioural strategies to manage HFNS. Assessments were done at baseline, 9 weeks, and 26 weeks after randomisation. The primary outcome was the adjusted mean difference in HFNS problem rating (1–10) between CBT and usual care groups at 9 weeks after randomisation. Analysis of the primary endpoint was done by modified intention to treat. The trial is registered, ISRCTN13771934, and was closed March 15, 2011.

**Findings:**

Between May 5, 2009, and Aug 27, 2010, 96 women were randomly allocated to group CBT (n=47) or usual care (n=49). Group CBT significantly reduced HFNS problem rating at 9 weeks after randomisation compared with usual care (mean difference −1·67, 95% CI −2·43 to −0·91; p<0·0001) and improvements were maintained at 26 weeks (mean difference −1·76, −2·54 to −0·99; p<0·0001). We recorded no CBT-related adverse events.

**Interpretation:**

Group CBT seems to be a safe and effective treatment for women who have problematic HFNS after breast cancer treatment with additional benefits to mood, sleep, and quality of life. The treatment could be incorporated into breast cancer survivorship programmes and delivered by trained breast cancer nurses.

**Funding:**

Cancer Research UK.

## Introduction

Breast cancer is the most common cancer in women in the UK and, as a result of improved early detection and treatment, more women are now living longer with or surviving the disease.^1^ Hot flushes and night sweats (HFNS) affect 65–85% of women treated for breast cancer, often occurring while women are still adjusting to the effects of cancer treatments. HFNS are more severe in this population than they are in healthy women and have a negative effect on quality of life, mood, and sleep.^2^, ^3^, ^4^ Chemotherapy or adjuvant endocrine treatments can compromise or lead to failure of ovarian function, resulting in rapid reduction of oestrogen concentrations, which induces or exacerbates HFNS. HFNS can in turn reduce adherence to endocrine therapy if left untreated.^5^, ^6^, ^7^

Hormone replacement therapy is an effective treatment for HFNS, but is generally contraindicated for patients with breast cancer because it can increase the risk of breast cancer recurrence, and HFNS can return in women who discontinue hormone replacement therapy.^8^ A Cochrane review of non-hormonal medical treatments concluded that clonidine (an antihypertensive drug), gabapentin (an anticonvulsant that works through an unknown mechanism), selective serotonin reuptake inhibitors (SSRIs), and serotonin-norepinephrine reuptake inhibitors (SNRIs) showed a mild to moderate effect on the frequency of HFNS in women with a history of breast cancer (reductions of between 15% and 58%; average 37%).^9^ Three trials done since the review was undertaken recorded similar reductions.^10^, ^11^, ^12^ Various side-effects of medical treatments were reported—eg, dry mouth, sleep problems, and nausea—and short follow-up limited conclusions about long-term outcomes.^9^ Some SSRIs (paroxetine and fluoxetine), but not all, might reduce the effectiveness of endocrine treatments.^13^ Evidence of improvements to quality of life is mixed; six of 12 studies of non-hormonal drugs reported no effect.^10^, ^11^, ^12^, ^14^ Moreover, many breast cancer survivors prefer to use non-medical treatments.^3^ For non-pharmacological therapies—eg, vitamins, magnetic devices, or acupuncture—the Cochrane review concluded that outcomes were either inconsistent or not statistically significant.^9^ Consequently, a need exists for safe, acceptable, and effective non-hormonal treatments that are free from side-effects to help these women to manage HFNS.^15^, ^16^

An intervention based on cognitive behavioural therapy (CBT) has been developed,^17^ including psychoeducation, paced breathing and relaxation, and CBT to help women to manage HFNS. This intervention has been shown to be acceptable to women and has shown promise in exploratory trials of one-to-one and group CBT.^17^, ^18^ The treatment is based on a model of the hypothesised causal mechanisms and maintaining factors of HFNS, which include anxiety, stress, embarrassment, negative beliefs, catastrophic thoughts, and avoidance behaviours.^19^, ^20^ In a pilot, uncontrolled trial of group CBT, 17 women who had been treated for breast cancer reported an average 49% reduction in HFNS problem rating and a 38% reduction in self-reported HFNS frequency.^18^

HFNS frequency has generally been considered to be the target of treatments, but studies have suggested that problem rating (ie, the extent to which HFNS are bothersome and interfere with life) should be the primary outcome in clinical trials because it is associated with help-seeking behaviour and quality of life to a greater extent than is frequency of HFNS.^21^, ^22^ Sternal skin conductance (SSC) monitoring is the most valid physiological measure of HFNS, but is rarely included in trials. Subjective and physiological measures assess different aspects of HFNS.^23^ MENOS 1 is a randomised controlled trial of group CBT compared with usual care with a 26-week follow-up. We hypothesised that participants would report less problematic HFNS and fewer HFNS, improved mood, sleep, and health-related quality of life after group CBT compared with individuals who received usual care. We measured subjective frequency and problem rating of HFNS and SSC monitoring of HFNS frequency, to establish whether the treatment affects physiological or psychological factors, or both.

## Methods

### Study design and participants

The study design is described in detail in the trial protocol.^20^ Briefly, recruitment took place between March 17, 2009, and Aug 27, 2010. Women attending breast or oncology clinics in southeast London, UK, were offered the trial by clinicians and research nurses. They could also apply for inclusion by responding to leaflets and posters displayed in clinics.

English-speaking women older than 18 years were eligible if they had had at least ten problematic HFNS per week (confirmed by a 2-week diary and a screening interview) for a duration of 2 months or more, had completed medical treatment for breast cancer (surgery, radiotherapy, or chemotherapy), and had no evidence of other cancers or metastases. Women taking adjuvant endocrine treatment were eligible. Because many women use treatments for HFNS but still have troublesome symptoms and seek further treatment, they were not excluded if they had been using the treatment consistently for 2 months or more, and planned to continue at the same dose during the trial. Those unable to attend sessions or who were seeking treatment for mood disorders rather than for HFNS were not eligible. All participants gave written, informed consent before taking part. Ethical approval was obtained from the UK NHS Research Ethics Committee.

### Randomisation and masking

Randomisation was done in cohort groups. After baseline assessment and receipt of consent from 12–20 participants, the trial clinical psychologist (MJS) sent participants' identification details to a programmer at the Clinical Trials Unit at King's College London, UK, for randomisation. The number of women in each cohort was determined on the basis of an estimation of an optimum of six to ten participants in group CBT. The programmer created a computer-generated randomisation sequence, allocating participants in a one-to-one ratio, stratified by age (<50 years, ≥50 years), with randomly varying block size. This process was repeated for each cohort group so that allocation did not affect the allocation sequence of subsequent cohorts. The clinical psychologist, who did the group CBT treatment, was sent the allocation results and then informed participants of their treatment allocation. Although neither participants nor the clinical psychologist could be masked to group allocation, researchers collecting outcome data and analysing results were masked. Specifically, at 9-week and 26-week assessments, women were met by a separate researcher who collected questionnaires and who also asked the women not to disclose their treatment allocation to the researcher who did the outcome assessments. To check whether the trial coordinator (EM) was successfully masked, after 9-week assessments for each cohort she noted which group she thought participants had been allocated to. Masking was successful, with the allocation of 51% of participants correctly identified. The trial statistician was masked to group identity until analyses were complete.

### Procedures

Assessments took place at baseline, 9 weeks after randomisation (typically 2 weeks after treatment), and 26 weeks after randomisation. About 2 weeks before participants attended a baseline assessment, a researcher explained the study, assessed eligibility by telephone, and posted written information and a 2-week diary to be updated on a daily basis to record HFNS frequency. At baseline assessment, eligibility was confirmed; participants were able to ask questions before signing a standard consent form. Women were asked about their breast cancer treatment history, menopause symptoms, and medical history. They completed a questionnaire covering demographic characteristics and baseline measures and were given a small SSC monitor and a magnetic event marker bracelet worn for 24 h. They were asked to indicate when they had an HFNS by passing the magnet over the monitor; they were encouraged to do usual activities and were shown how to remove the monitor to shower or bathe.

Participants allocated to receive group CBT attended a 90 min session every week for 6 weeks, between June, 2009, and October, 2010 (panel 1). A treatment manual was produced in advance of the study, which contained detailed session content, presentation slides and handouts, and notes for facilitators. A clinical psychologist was trained to deliver the sessions with the help of an assistant (five assistants took part over the course of the study). All sessions were audio taped, then 10% were randomly selected (with a computer-generated random number sequence) and a psychologist (MSH) experienced in cognitive behavioural therapy for HFNS, rated them for adherence to the treatment manual, by indicating on coding sheets the extent to which the group leader covered each topic. Coding sheets included specific components of the intervention (eg, reviewing homework, providing information about the role of stress, demonstrating paced breathing in the session, group discussion of behaviours relating to HFNS) developed for the trial (appendix).

Panel 1 **Intervention content**

**Group cognitive behavioural therapy (CBT)**

The group CBT was psycho-educational, structured, and interactive with presentations, group discussion, handouts, and weekly homework. Paced breathing and relaxation were practised at each session and participants were given a relaxation and paced breathing audio CD to practise at home daily and during hot flushes and night sweats (HFNS). Women recorded their HFNS in weekly diaries.

Session one introduced the cognitive behavioural model including physiological, cognitive, behavioural, and emotional components of HFNS; provided information about the physiology of HFNS; and introduced paced breathing. Participants described their experiences of HFNS in the context of breast cancer, outlined their goals for treatment, discussed and recorded particular triggers of hot flushes, and practised relaxation.

Session two focused on the role of stress in potentiating HFNS and CBT strategies for the reduction of stress and anxiety. Paced breathing was introduced and practised as homework.

Session three focused on cognitive (eg, catastrophic thinking and overly negative beliefs about hot flushes) and behavioural reactions (eg, avoidance of activities) to hot flushes. Participants planned ways to manage flushes in social situations and paced breathing to manage flushes was introduced. They practised paced breathing at onset of a flush and implemented cognitive behavioural strategies to manage hot flushes as homework. They also completed sleep diaries in preparation for the sleep sessions.

Session four focused on understanding night sweats and improving sleep habits and the application of behavioural strategies to reduce wakefulness after night sweats. Participants identified ways in which they could change sleep habits, which they implemented as homework.

Session five focused on the cognitive component of sleep problems, including sleep-related anxieties or general worries leading to further wakefulness. CBT strategies were developed for the management of night sweats.

Session six was a review session and participants made action plans to maintain cognitive and behavioural changes, including dealing with setbacks.

**Usual care**

All women had completed active breast cancer treatment and were typically followed up every 6 months by an oncologist or clinical nurse specialist, with additional appointments as needed. Additionally, those treated in UK National Health Service hospitals in southeast London were offered telephone support as part of the cancer survivorship programme. Women were sent an information leaflet produced by Breast Cancer Care and offered telephoned support every 2 weeks (average seven telephone calls, maximum ten). Nurses gave information about HFNS, advised on treatment options and practical ways of symptom management, and offered instructions for paced breathing and relaxation. 77 (80%) participants had access to the survivorship programme. The remainder were no longer attending follow-up appointments at the hospital (n=7), or were not treated at hospitals in the southeast London cancer network (n=12). Therefore, individuals in the usual care group received information and had access to high quality support services.

All participants received usual care—they had access to clinical specialists and cancer support services, either through routine follow-up appointments or as part of a breast cancer survivorship programme in southeast London (panel 1). At the second assessment (9 weeks after randomisation) they repeated the baseline questionnaires and 24-h SSC HFNS monitoring, and documented any health and lifestyle changes as well as use of services and treatments in the previous 9 weeks (the latter was used to index usual care in both treatment groups). At 26 weeks after randomisation, questionnaires were sent containing the same measures; face to face or telephone interviews were done by an independent psychologist after final measures were completed to obtain women's responses to being in the trial and their views about the treatment (for those in the CBT group). HFNS treatments used during the trial were recorded for each participant at each timepoint. Follow-up was completed by March 15, 2011.

### Outcomes

The primary outcome was the HFNS problem rating (hot flush rating scale^24^) at 9 weeks after randomisation. Problem rating—ie, the extent to which symptoms are bothersome and interfere with life—was chosen, before the trial began,^20^ as the primary outcome measure because problem rating, rather than frequency, is associated with help-seeking and quality of life, and it has been recommended as the most appropriate patient-reported outcome measure in clinical trials of HFNS treatments.^21^, ^22^ Problem rating and severity tend to be associated with each other—neither are strongly associated with frequency of HFNS.^21^

Secondary outcomes included HFNS problem rating at 26 weeks, and HFNS frequency, mood, and health-related quality-of-life measures at 9 weeks and 26 weeks after randomisation. Problem rating of HFNS (hot flush rating scale^24^) was calculated as the mean of three 10-point items that assess the extent to which symptoms are problematic and interfere with daily life; 10 indicates most problematic HFNS. A difference of two points or more is regarded as clinically relevant.^17^, ^18^, ^25^ The scale has good internal consistency (Cronbach α=0·9) and test-retest reliability (*r*=0·8). HFNS frequency was measured with the HFNS frequency subscale (total number of HFNS reported in the past week) of the Hot Flush Rating Scale.^24^ This subjective HFNS frequency measure correlated with daily diary recordings of HFNS in a previous study (*r*=0·9, p<0·0001).^24^

SSC monitoring and participant-reported flushes were assessed over a 24-h monitoring period (with the Bahr SSC monitor [Simplex Scientific; Middleton, WI, USA]). A 6-cm by 6-cm monitor measured SSC every 10 s by passing an electric current across two electrodes attached to the sternal region of the chest. Women also indicated occurrence of a hot flush by passing a magnetic event marker over the monitor, which recorded the time. HFNS were defined by increases of 2 μS or more occurring within 30 s, and not within 15 min duration since the previous HFNS. This is the standard criteria used for SSC monitoring validated with ambulatory and laboratory equipment.^23^ The sum of physiological and participant-reported (event-marked) HFNS were extracted for analysis. When less than 24 h of data were available, number of HFNS was adjusted so that it was equivalent to the rate of HFNS per 24 h.

The General Health Survey Short Form 36 (SF-36)^26^ was used to measure dimensions of health-related quality of life (general health, mental health, physical role limitation, emotional role limitation, physical functioning, social functioning, vitality, and bodily pain); scores range from 0 to 100 (a higher score indicates better health).

The Women's Health Questionnaire (WHQ),^27^ a measure of physical and emotional symptoms designed for and standardised on women aged 45–65 years, is widely used in studies of menopausal women and has high internal reliability. The WHQ is comprised of 37 items that assess, for example, an individual's depressed mood, anxiety or fears, and problems sleeping. Participants rate each item on a 4-point scale, according to the extent to which they are experiencing each item, and subscale scores are calculated, ranging from 0–1 (higher scores indicate poorer wellbeing). The depressed mood subscale has concurrent validity with the GHQ and the WHQ has good internal reliability for subscales (Cronbach α 0·70–0·84) and test-retest reliability (0·78–0·96). Five subscales were included: depressed mood, anxiety or fears, sleep problems, memory or concentration, and somatic symptoms. We also included measures of process variables including beliefs, behaviours, and stress, which are described elsewhere.^20^

Adherence to group CBT was measured by the number of sessions attended and the number of times that a participant practised relaxation or paced breathing each week. Number and type of adverse events were examined in both groups to assess treatment safety in accordance with the protocol. The trial data management committee reviewed adverse events and assessed whether they were related to the intervention. Recurrence of breast cancer, skin irritation due to the hot flush monitor, and clinically significant deterioration in mood were identified as possible expected unrelated adverse events, and clinically significant deterioration in mood as a result of group CBT was identified as a potential treatment-related adverse event. The trial clinical psychologist recorded incidents of mood deterioration and referred patients for treatment, as appropriate. Recurrence of breast cancer or secondary cancers were classified as expected but unrelated serious adverse events.^28^

### Statistical analysis

We calculated that a sample size of 96 women was needed to provide 90% power to detect a two-point difference (SD 2·4; standardised effect size 0·8) in mean HFNS problem rating for the comparison of CBT to usual care at 9 weeks after randomisation. This sample size allowed for 20% loss to follow-up, a variance inflation factor of 1·49 (intraclass correlation 0·07,^29^ eight participants per group) to power for expected clustering of outcomes, and an HFNS problem rating baseline-to-outcome correlation of 0·4 on analysis of covariance with two-sided 5% significance levels.

The statistical analysis plan was finalised and approved by the trial team before completion of data collection. p values of less than 0·05 were regarded as significant for all analyses. Analyses were based on modified intention-to-treat sample (excluding those who contributed no data). We assessed the primary outcome with a linear mixed model; covariates were treatment group, baseline HFNS problem rating score, the stratification factor age (as a dichotomous category, split at 50 years), and time. A time-by-treatment interaction term was included to allow estimates at the individual timepoints to be summarised. The model for problem rating incorporated two levels of random intercepts by participant and cohort group. The modelling was done with the assumption that data were missing at random and predictors of missing data (treatment group) were included in the modelling. We used a logistic model to assess predictors of missing data, and to examine all baseline clinical and demographic variables. Categorical outcomes, including adverse events were compared with Fisher's exact test. We also analysed secondary outcomes with mixed linear regression models with random participant and cohort group intercepts and a time-by-treatment interaction term; covariates in the model were treatment group, baseline value of outcome, the stratification factor age, and time. Proportions and associated CIs were estimated from Wilson's binomial distribution. Results from all analyses were summarised at 9 weeks and 26 weeks with two-sided 95% CIs. We used Stata (version 11.0) for all statistical analyses. The trial is registered with Current Controlled Trials, number ISRCTN13771934.

### Role of the funding source

The sponsor of the study had no role in the study design, data collection, data analysis, data interpretation, or writing of the report. The corresponding author had full access to all the data in the study and had final responsibility for the decision to submit for publication.

## Results

**Figure 1:**
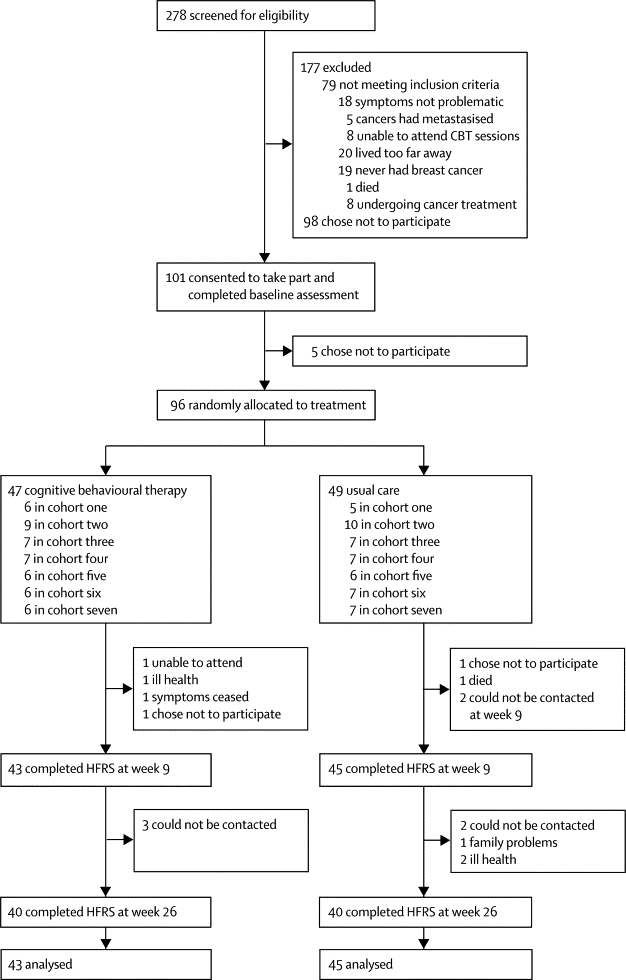
Trial profile CBT=cognitive behavioural therapy. HFRS=hot flush rating scale.

We randomly allocated 96 women to treatment between May 5, 2009, and Aug 27, 2010, most of whom remained in the trial (figure 1). Withdrawal rates were much the same in both treatment groups (p=0·99 at 9 weeks; p=0·79 at 26 weeks), as were baseline demographic and clinical characteristics of participants (table 1). On starting the trial, HFNS were frequent and problematic with a mean of 69 per woman (SD 39) occurring per week for an average of 2 years, with a mean problem rating of 6·3 out of 10 (table 1). 20 women were taking herbal remedies or complementary therapies for HFNS at baseline, all of whom were symptomatic. 13 women were taking non-hormonal prescribed drugs (table 1): hormone replacement therapy (one woman in each group), SSRI (two women in the CBT group and three in the usual care group), gabapentin (one woman in each group), clonidine (two women in the CBT group), gabapentin plus SSRI (one woman in the CBT group), and clonidine plus SSRI (one woman in the usual care group).

**Table 1:** Demographic and clinical baseline characteristics

		**Cognitive behavioural therapy (n=47)**	**Usual care (n=49)**
Age at randomisation (years; mean [SD])		53·16 (8·10)	54·05 (7·76)
Individuals younger than 50 years		15 (32%)	17 (35%)
Ethnic origin			
	White	42 (89%)	40 (82%)
	Black	4 (9%)	5 (10%)
	Other	1 (2%)	4 (8%)
Number of women who have had children		29 (62%)	31 (63%)
Married or living with partner		29 (62%)	28 (57%)
Educated beyond 16 years of age		30 (64%)	33 (67%)
Employed		30 (64%)	32 (65%)
Mean body-mass index (kg/m^2^; SD)		27·13 (5·30)	27·51 (6·90)
Premenopausal before diagnosis		24 (51%)	24 (49%)
Perimenopausal before diagnosis		9 (19%)	8 (16%)
Postmenopausal before diagnosis		12 (25%)	16 (33%)
Time since breast cancer diagnosis (months; mean [SD])		47·75 (53·38)	31·08 (30·63)
Treatment history			
	Surgery	46 (98%)	49 (100%)
	Chemotherapy	26 (55%)	37 (76%)
	Radiotherapy	36 (77%)	41 (84%)
Taking endocrine treatment at baseline		34 (72%)	36 (73%)
Chronicity of HFNS (months; mean [SD])		23·53 (34·46)	28·48 (43·11)
Taking a prescribed drug for HFNS at baseline		7 (15%)	6 (12%)
Baseline HFNS problem-rating (mean [SD])		6·52 (2·43)	6·12 (2·02)
Baseline HFNS frequency (number per week; mean [SD])		72·84 (37·89)	66·34 (40·18)

Data are n (%), unless otherwise stated. HFNS=hot flushes and night sweats.

**Figure 2:**
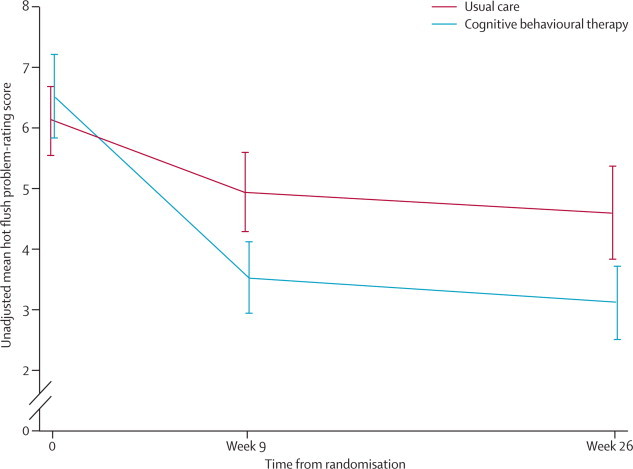
Changes in problem-rating scores for hot flushes and night sweats Error bars show 95% CIs.

Problem rating scores at 9 weeks and 26 weeks were lower in the CBT group than they were in the usual care group (table 2). There was a significant difference between groups in the primary outcome—HFNS problem rating at 9 weeks following randomisation (adjusted mean difference of −1·67, 95% CI −2·43 to −0·91; p<0·0001) with a greater reduction from baseline in problem rating in the CBT group compared to the usual care group. At 9 weeks, the change in problem rating from baseline was −3·05 (SD 2·3) in the CBT group, compared with −1·06 (SD 1·7) in usual care group, equating to a 46% reduction in the CBT group and a 19% reduction in the usual care group. At 26 weeks, the problem rating had decreased from baseline by 52% (mean change −3·39, SD 2·3) in the CBT group and by 25% (mean change −1·26, SD 2·2) in the usual care group (table 2; figure 2).

**Table 2:** Hot flush and night sweats problem-rating scores

	**Cognitive behavioural therapy (mean [SD])**	**Usual care (mean [SD])**	**CBT***vs*usual care*(adjusted mean difference [SE, 95% CI; p value])
Baseline	6·52 (2·43)	6·12 (2·02)	..
9 weeks	3·53 (1·98)	4·95 (2·24)	−1·67 (0·39, −2·43 to −0·91; <0·0001)
26 weeks	3·13 (1·94)	4·60 (2·48)	−1·76 (0·40, −2·54 to −0·99; <0·0001)

* Adjusted analysis used cohort number as a random effect and a covariate for the binary age stratification factor.

We recorded no significant difference between groups in HFNS frequency, or in the hot flush frequency and night sweat frequency subscales when analysed separately, at 9 weeks or 26 weeks (table 3 and appendix). Compared with baseline, both groups reported non-significantly fewer HFNS at 9 weeks (21% in the CBT group and 24% in the usual care group) and 26 weeks (38% in both groups). We recorded little change in 24-h rate of HFNS at 9 weeks, measured either by sternal skin conductance or participant-reported event markers; all frequency measures showed large variability in response (table 3).

**Table 3:** Effect of treatment on hot flushes and night sweats

	**Cognitive behavioural therapy (mean [SD])**	**Usual care (mean [SD])**	**Adjusted mean (difference [SE])**	**95% CI**
**Total HFNS frequency**				
Baseline	72·84 (37·89)	66·34 (40·18)	..	..
9 weeks	57·72 (43·73)	50·31 (32·44)	2·89 (7·03)	−10·89 to 16·67
26 weeks	45·33 (45·93)	41·37 (28·90)	−5·21 (7·39)	−19·69 to 6·27
**Hot flush frequency**				
Baseline	58·64 (32·16)	52·98 (37·93)	..	..
9 weeks	45·60 (38·00)	36·76 (29·18)	6·63 (6·51)	−6·13 to 19·40
26 weeks	37·46 (41·41)	30·77 (25·40)	0·95 (6·76)	−12·29 to 14·20
**Night sweats frequency**				
Baseline	16·31 (14·84)	13·50 (10·13)	..	..
9 weeks	12·12 (9·93)	13·30 (8·69)	−2·09 (1·93)	−5·86 to 1·69
26 weeks	8·48 (9·13)	10·67 (9·97)	−3·31 (2·03)	−7·28 to 0·66
**24 h sternal skin conductance frequency (n=89)**				
Baseline	9·38 (8·83)	11·09 (9·31)	..	..
9 weeks	12·05 (8·03)	10·23 (6·80)	2·29 (1·48)	−0·62 to 5·19
26 weeks	..	..	..	..
**24 h event-marker frequency (n=89)**				
Baseline	10·83 (8·96)	9·11 (8·33)	..	..
9 weeks	10·00 (7·84)	7·76 (5·10)	1·91 (1·51)	−1·04 to 4·87
26 weeks	..	..	..	..
**WHQ depressed mood**				
Baseline	0·23 (0·26)	0·31 (0·27)	..	..
9 weeks	0·13 (0·16)	0·28 (0·24)	−0·14 (0·05)*	−0·23 to −0·06
26 weeks	0·13 (0·19)	0·28 (0·26)	−0·13 (0·05)*	−0·22 to −0·05
**WHQ somatic symptoms**				
Baseline	0·56 (0·26)	0·55 (0·25)	..	..
9 weeks	0·44 (0·24)	0·46 (0·24)	−0·08 (0·06)	−0·21 to 0·04
26 weeks	0·45 (0·23)	0·53 (0·23)	−0·03 (0·06)	−0·16 to 0·09
**WHQ anxiety or fears**				
Baseline	0·34 (0·25)	0·45 (0·30)	..	..
9 weeks	0·23 (0·27)	0·40 (0·33)	−0·12 (0·06)†	−0·24 to −0·01
26 weeks	0·24 (0·31)	0·39 (0·31)	−0·10 (0·06)	−0·21 to 0·01
**WHQ sleep problems**				
Baseline	0·63 (0·30)	0·72 (0·29)	..	..
9 weeks	0·37 (0·31)	0·65 (0·32)	−0·26 (0·07)‡	−0·39 to −0·12
26 weeks	0·43 (0·37)	0·61 (0·34)	−0·16 (0·07)†	−0·29 to −0·02
**WHQ memory and concentration**				
Baseline	0·75 (0·34)	0·72 (0·36)	..	..
9 weeks	0·59 (0·36)	0·70 (0·32)	−0·14 (0·06)†	−0·27 to −0·02
26 weeks	0·51 (0·37)	0·62 (0·36)	−0·14 (0·06)†	−0·26 to −0·02
**SF-36 physical functioning**				
Baseline	66·17 (22·89)	74·89 (22·27)	..	..
9 weeks	75·38 (24·24)	79·23 (21·96)	4·76 (3·47)	−2·03 to 11·56
26 weeks	74·13 (24·96)	73·88 (27·37)	8·86 (3·46)†	2·09 to 15·64
**SF-36 role—physical**				
Baseline	53·72 (43·29)	49·46 (40·31)	..	..
9 weeks	60·00 (40·35)	60·90 (39·65)	−1·09 (8·14)	−17·03 to 14·85
26 weeks	55·77 (43·10)	51·92 (44·20)	2·63 (8·17)	−13·39 to 18·65
**SF-36 bodily pain**				
Baseline	46·15 (22·73)	52·99 (21·64)	..	..
9 weeks	53·68 (23·98)	52·16 (22·57)	6·35 (4·20)	−1·89 to 14·59
26 weeks	51·00 (22·50)	46·58 (22·18)	9·85 (4·20)†	1·61 to 18·09
**SF-36 general health**				
Baseline	48·10 (15·94)	49·32 (16·77)	..	..
9 weeks	51·84 (14·58)	47·68 (17·81)	5·36 (2·53)†	0·40 to 10·32
26 weeks	50·34 (15·42)	44·98 (19·83)	7·11 (2·53)*	2·15 to 12·07
**SF-36 vitality**				
Baseline	35·33 (16·10)	38·13 (16·50)	..	..
9 weeks	39·63 (15·23)	38·89 (17·79)	3·57 (2·92)	−2·16 to 9·29
26 weeks	40·31 (17·48)	38·96 (15·72)	3·46 (2·92)	−2·26 to 9·17
**SF-36 social functioning**				
Baseline	67·02 (31·43)	71·20 (28·00)	..	..
9 weeks	75·30 (25·39)	75·64 (25·96)	3·01 (4·97)	−6·72 to 12·74
26 weeks	77·50 (27·18)	62·81 (29·48)	16·29 (4·96)*	6·57 to 26·00
**SF-36 role—emotional**				
Baseline	67·39 (42·45)	55·56 (42·64)	..	..
9 weeks	75·61 (38·02)	64·10 (40·02)	7·48 (7·97)	−8·14 to 23·10
26 weeks	73·50 (37·60)	60·68 (42·49)	7·66 (8·07)	−8·14 to 23·47
**SF-36 mental health**				
Baseline	67·57 (17·89)	62·52 (17·37)	..	..
9 weeks	74·63 (14·22)	66·46 (14·20)	6·03 (2·95)†	0·24 to 11·81
26 weeks	70·70 (19·24)	64·5 (16·06)	3·86 (2·96)	−1·94 to 9·65

For the *Women's Health Questionnaire* (WHQ),^27^ norms for women aged 45–65 years (n=682) are as follows (mean [SD]): depressed mood, 0·22 (0·23); somatic symptoms, 0·39 (0·25); anxiety or fears, 0·35 (0·28); sleep problems, 0·45 (0·36); and memory and concentration, 0·47 (0·36). HFNS=hot flushes and night sweats. SF-36=General Health Survey Short Form 36.^26^

* p<0·01.

† p<0·05.

‡ p<0·0001.

Women in the CBT group reported less depressed mood and fewer sleep problems at week 9 and week 26 compared with those in the usual care group (table 3 and appendix). Women receiving CBT also reported less anxiety than did women in the usual care group at 9 weeks, but this difference was not statistically significant at 26 weeks (table 3). We recorded small improvements in memory and concentration of participants in the CBT group compared with those in the usual care group, but no statistically significant differences in somatic symptoms (table 3). At 26 weeks, women in the CBT group reported significantly better social functioning, physical functioning, and improved general health (at both 9 weeks and 26 weeks) than did women in the usual care group, according to the SF-36 assessments (table 3). Compared with women in the usual care group, women in the CBT group reported better mental health at 9 weeks and less bodily pain at 26 weeks (table 3). We recorded no significant differences between the groups in emotional role functioning or vitality subscales (table 3).

**Figure 3:**
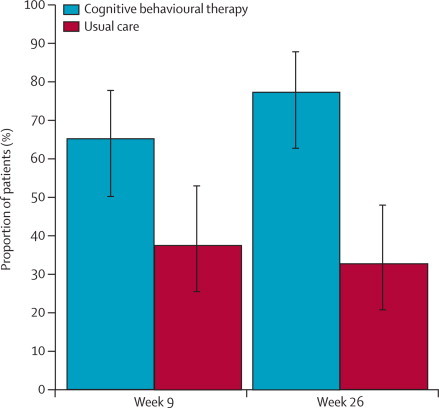
Proportion of patients with a reduction of two or more points in the hot flush problem-rating scale from baseline Estimates are unadjusted. Error bars are 95% CIs.

Although we did not record a mean two-point difference between treatment groups (figure 3), a greater percentage of individuals had reached this two-point threshold in the CBT group than in the usual care group at both 9 weeks (65% [95% CI 50–78] *vs* 38% [25–52]) and 26 weeks (78% [62–88] *vs* 33% [20–48]). This exploratory analysis suggests that improvement from baseline was clinically better in the CBT group than it was in the usual care group (figure 3).

In the 10% of sessions assessed for quality assurance, CBT was delivered according to the treatment manual, with 98–100% adherence. Training and clinical supervision were provided by a consultant clinical psychologist (MSH) throughout the study. Participant adherence to treatment was good; 39 (91%) of 43 participants who received CBT attended at least four of six sessions, and relaxation or paced breathing was practised, on average, 29 times each week (SD 26·93).

Individuals taking prescribed drugs for HFNS tended to continue them throughout the trial. Exceptions were one participant who stopped taking fluoxetine (CBT group), and one woman who stopped taking clonidine (usual care group). Three women started homoeopathy or herbal remedies (one in the CBT group, two in the usual care group), and one woman in the usual care group started acupuncture. When the adjusted primary analyses were repeated excluding these five participants, the estimates and CIs remained within the same margins (week 9 hot flush problem rating, CBT *vs* usual care mean difference −1·58, 95% CI −2·38 to −0·79; p<0·0001; week 26 hot flush problem rating, CBT *vs* usual care mean difference −1·77, −2·59 to −0·95; p<0·0001).

We recorded six adverse events, three in the CBT group and three in the usual care group. One participant died of an unrelated disorder before the treatment phase (usual care group), we recorded two reoccurrences of breast cancer (one in each group), and one woman reported low mood unrelated to the trial (CBT group). Two women (both in the usual care group) had mild reactions to the hot flush monitor (a reaction to the adhesive used on the electrode patch). None of the adverse events were related to CBT.

## Discussion

Our findings suggest that CBT is safe, acceptable, and effective in helping women to manage hot flushes and night sweats after breast cancer treatment, with additional benefits to mood, sleep, and some aspects of quality of life. After group CBT, women reported less problematic HFNS than did those receiving usual care, and these improvements were maintained at both the 9-week and 26-week follow-up. Although the pre-specified endpoint of a between-group difference of 2 points on the problem-rating scale was not met, there was a statistically significant improvement in the CBT group, and a large proportion of women (78%) who had received CBT achieved a reduction of 2 points compared with the usual care group (33%) at 26 weeks. However, we recorded no significant difference in the frequency of HFNS between the two groups at either 9 weeks or 26 weeks. Similarly, we recorded no significant differences between groups in 24 h rate of HFNS after the treatment phase, either physiologically (SSC) defined or participant recorded (event monitor). Nevertheless, the effects of CBT on HFNS problem rating were substantial and robust. Most women participating in group CBT reported reductions in problem rating that are regarded as clinically important, and there were sustained significant differences in problem rating between CBT and usual care. These differences were not unduly affected by outliers or other influential points (ie, the average group changes were significant), and the sample contained more precise standard deviations than we expected.

Additionally, group CBT provided sustained benefits to depressed mood and sleep and some improvements in dimensions of quality of life. At baseline, on average, participants had higher scores for sleep problems, somatic symptoms, and memory and concentration (WHQ) compared with norms for healthy women (table 3 and data not shown).^27^ According the the SF-36 scale, they had lower scores (lower quality of life) than did healthy women, especially for physical role functioning, general health, vitality, and bodily pain. The significant improvements in mood and sleep, as well as memory and concentration, after CBT are clinically important because difficulties with mood sleep and cognitive functions are commonly reported by patients with breast cancer, particularly by those with HFNS.^30^ Moreover, the improvement in social functioning after CBT is relevant because women report finding hot flushes especially difficult to deal with at work and in other social situations.^18^ Although randomised controlled trials of non-hormonal drugs for HFNS have shown moderate reductions in HFNS frequency, they have recorded little evidence of improved quality of life.^9^ Similarly, some studies show only weak relations between frequency of HFNS and quality of life.^21^, ^22^

The results of our study are very similar to the preliminary findings of a parallel trial of CBT for HFNS in a sample of healthy women (MENOS2).^31^ This consistent pattern of results suggests that the CBT might work by affecting symptom perception and cognitive appraisal of HFNS and possibly mood, rather than physiological mechanisms of HFNS, such as temperature regulation or the thermoneutral zone in the hypothalamus.^19^ Further analyses of moderators and mediators of HFNS are planned to clarify this issue through identification of the underlying causal pathways to improvement on the hot flush problem rating scale.^20^ For example, changes in sleep after treatment might partly mediate the improvements in mood and quality of life. We postulate that hot-flush beliefs and behaviours, as well as mood and sleep, will act as potential mediators. For the moderator analysis, baseline clinical and demographical characteristics, such as anxiety and depression, will be considered alongside factors such as drug use and health behaviours. Further studies should examine particular components of the intervention to test these analyses experimentally.

We postulated that CBT would reduce HFNS frequency to a greater extent than would usual care, especially because paced breathing has been shown to reduce HFNS frequency measured both physiologically and subjectively.^32^ Women receiving CBT reported practising paced breathing daily. Paced breathing, once learnt, is practised for 2–5 min throughout the day and at the onset of a hot flush, so an average of 29 times per week is a realistic rate at which to practise. However, participants in both groups reported improvements in HFNS frequency. Women in the usual care group did receive psychoeducation, advice (which included paced breathing), and nursing support, which could have led to reductions in the usual care group, although we did not record paced breathing or other techniques done by these women. CBT treatment effects could have been attributable to the placebo effect of additional attention in the group CBT. However, we controlled for usual care and assessment effects and we should gain some insight into the placebo effects of usual care through the planned mediator and moderator analysis.^20^ Further research could compare different types of control conditions with differing levels of information, advice, and support. The group CBT programme should be generalisable to other breast cancer settings because it is delivered in a hospital setting, is done by use of easily transferable guidelines (the treatment manual), and adherence to the guidelines was high. Drop-out rates were low, and the preliminary results of qualitative interviews done at the end of the trial suggest that the CBT was highly acceptable. The cost of delivering the treatment needs to be assessed, with a need for comparison with non-hormonal drugs. The most cost-effective method of delivering the group CBT would probably be to include it as part of survivorship support programme, delivered by trained and supervised breast-care nurses.

To date, the best available treatments have been non-hormonal drugs including SSRI and SNRIs, clonidine, and gabapentin.^9^ These treatments have produced moderate reductions in HFNS frequency (averaging 37% across trials^9^), but have had little effect on quality-of-life measures. By contrast our findings suggest that both CBT and usual care resulted in a 38% reduction in frequency, and compared with the usual care group, those who received CBT showed statistically significant and lasting reductions in problem rating and improvements in quality of life. CBT could, therefore, be an important alternative or additional treatment option for patients with breast cancer (panel 2).

Panel 2 **Research in context**

**Systematic review**

We searched OvidSP using a combination of the terms cognitive “behavio*”, “hot flush*”, “hot flash*”, “menopaus*”, and “breast cancer”. We found three relevant studies. One study was the MENOS1 single-group pilot trial,^18^ in which weekly 1·5 h sessions of cognitive behavioural therapy (CBT) for 6 weeks resulted in an average 49% reduction in problem rating and 38% reduction in hot flushes and night sweats (HFNS) frequency, maintained at 3 months' follow-up. A larger randomised controlled trial of CBT and exercise,^33^ which used the group protocol manual developed for MENOS 1,^18^ reported preliminary findings in which frequency and problem-rating reduced compared with usual care, although detailed findings are not available. In another single-group trial,^34^ an instructional DVD of paced breathing and distraction used over the course of 1 week resulted in small reductions in bothersomeness and interference of HFNS and no change in physiologically measured hot flush frequency. CBT shows some promise but adequately powered randomised controlled trials are needed.

**Interpretation**

Our findings show that group CBT can reduce the effect of hot flushes and night sweats for women who have had breast cancer treatment. These reductions were sustained and associated with improvements in mood, sleep, and quality of life. Group CBT seems to be a safe, acceptable, and effective treatment option which can be incorporated into breast cancer survivorship programmes and delivered by trained breast cancer nurses.

Limitations of this study include the frequency measures, which had high variability. The main subjective measure (hot flush rating scale) is retrospective—daily diary measures might be more reliable. However, findings from another study show evidence of the validity of this scale compared with daily diary measures.^24^ Estimation of the frequency of night sweats with the hot flush rating scale can be more difficult because women do not report night sweats that they sleep through, but this limitation is common to all self-report measures. The physiological measure was used for 24 h at baseline and at 9 weeks after randomisation; in view of the variability of this measure, future studies might include a longer time of SSC monitoring and also include this measure at follow-up assessments. Additionally, we did not control for all potential confounding factors that could have an effect on menopausal symptoms, such as the use of drugs to manage HFNS and the use of adjuvant hormone therapy. Health behaviours might also have affected HFNS and other secondary outcomes, but were not controlled for in the analyses (eg, caffeine intake). Finally, we do not know whether HFNS were caused by breast cancer treatments or whether women were naturally menopausal when they had breast cancer. However, treatment options are still restricted for these women, so a need still exists for non-hormonal interventions.

Our findings suggest that this cognitive behavioural treatment, designed to be delivered by trained health professionals such as breast-care nurses, has the potential to improve long-term health outcomes for patients with breast cancer, and could be incorporated into breast cancer survivorship programmes.
