# Supplementary material for: Cognitive behavioural treatment for women who have menopausal symptoms after breast cancer treatment (MENOS 1): a randomised controlled trial
Source: Lancet Oncol. 2012 Mar;13(3):309–18. doi: 10.1016/S1470-2045(11)70364-3 (PMC3314999; doi:10.1016/S1470-2045(11)70364-3)
Supplement: Supplementary appendix [file mmc1.pdf]

THE LANCET Oncology

Supplementary appendix

This appendix formed part of the original submission and has been peer reviewed. We post it as supplied by the authors.

Supplement to: Mann E, Smith MJ, Hellier J, et al. Cognitive behavioural treatment for women who have menopausal symptoms after breast cancer treatment (MENOS 1): a randomised controlled trial. *Lancet Oncol* 2012; published online Feb 15. DOI:10.1016/S1470-2045(11)70364-3.

Web Appendix

Figure 1: Unadjusted means and confidence intervals for weekly frequency of hot flushes at baseline, 9 and 26 weeks post-randomisation

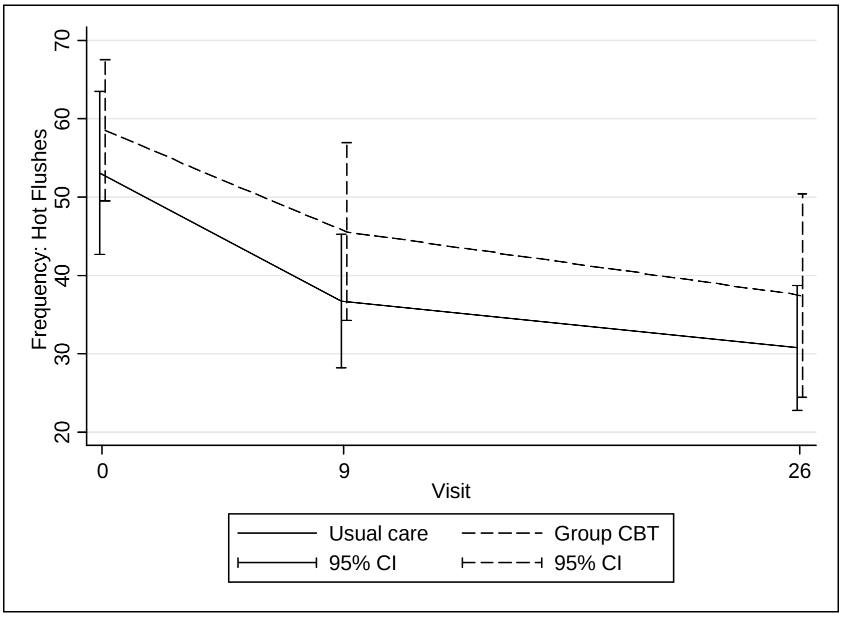

Figure 2: Unadjusted means and confidence intervals for weekly frequency of night sweats at baseline, 9 and 26 weeks post-randomisation

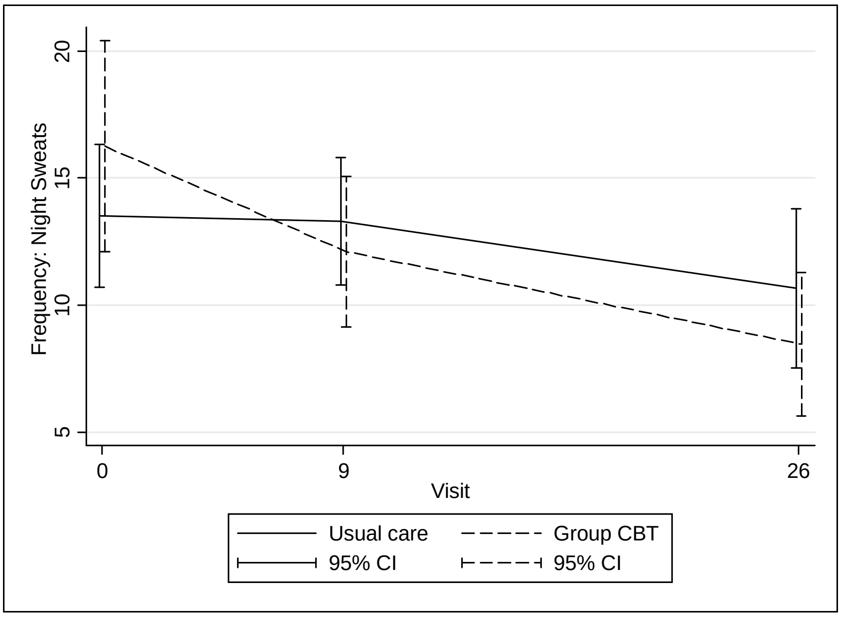

Figure 3: Unadjusted means and confidence intervals for WHQ depressed mood at baseline, 9 and 26 weeks post-randomisation

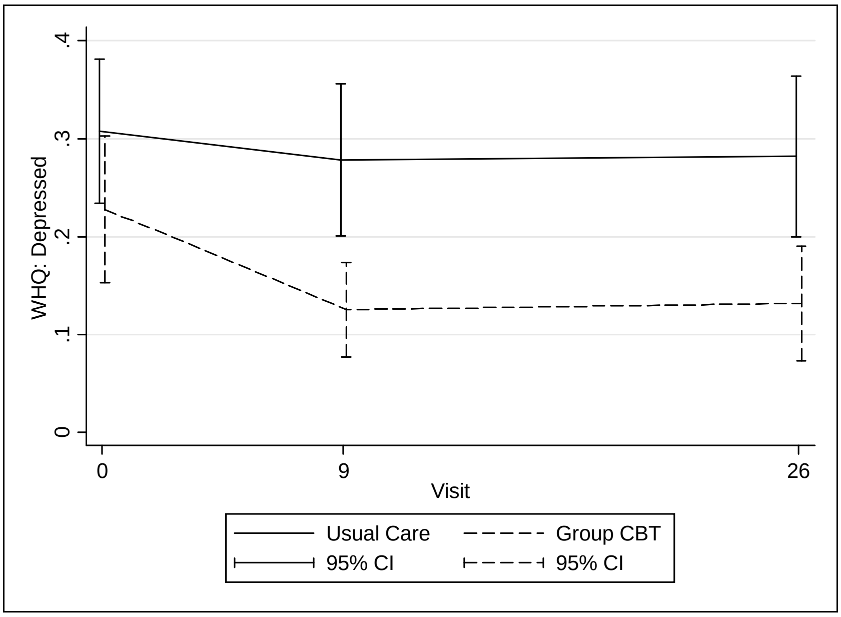

Figure 4: Unadjusted means and confidence intervals for WHQ Sleep problems at baseline, 9 and 26 weeks post-randomisation

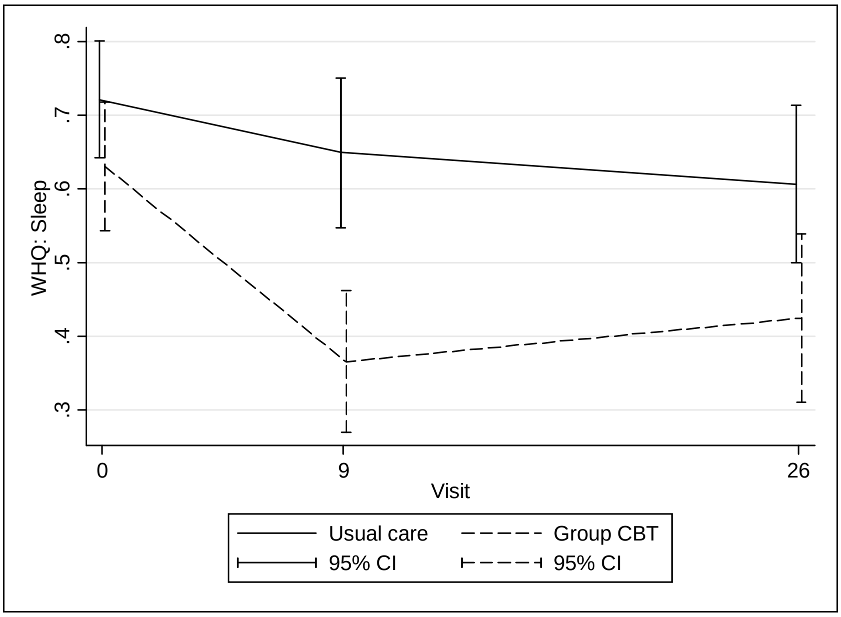

Quality Assurance for Group CBT intervention

Independent Session Assessment
Session One MENOS 1

Rater ……………………. Group therapist…….

Date of group………………….

**Session aims**	**Comments**
Introduce group members	
Outline group ground rules	
Create a supportive group atmosphere in which women are encouraged to participate	
Provide clear information about the physiology of hot flushes and night sweats? (i.e. thermo-neutral zone)	
Provide clear information about the cognitive behavioural model of hot flushes and night sweats?<br>(E.g. Link thoughts to feelings)	
Deliver main message – stress can affect hot flushes	
Provide clear information about role of triggers	
Practice relaxation during the session	
Elicit and respond to questions appropriately	
Outline and encourage homework and give out homework sheets and questionnaires for the next week	
Adopt empathic, positive and supportive therapeutic style	

Additional Comments:

Signed: Date:

Quality Assurance for Group CBT intervention

Independent Session Assessment
Session Two MENOS 1

Rater …………………….   Group therapist…….

Date of group………………….

**Session aims**	**Comments**
Review homework on precipitants	
Group discussion of precipitant modification	
Reminder of role of stress in hot flushes	
Provide clear information about stress to include fight or flight response and cognitive factors	
Group to consider their own cognitive and behavioural aspects of stress	
Deliver information on cognitive interventions for stress	
Deliver information on behavioural interventions for stress	
Group members to generate own stress plan for homework task	
Practice relaxation during the session	
Elicit and respond to questions appropriately	
Outline and encourage homework and give out homework sheets and questionnaires for the next week	
Adopt empathic, positive and supportive therapeutic style	

Additional Comments:

Signed: Date:

Quality Assurance for Group CBT intervention

Independent Session Assessment
Session Three MENOS 1

Rater ……………………. Group therapist…….

Date of group………………….

Session aims	Comments
Review homework on precipitants and wellbeing goals	
Reminder of 4 factor model and role of thoughts	
Provide clear information on nature of thoughts in hot flushes	
Provide clear information on types of thinking linked to increased distress including social situations and control	
Deliver information on addressing these types of thoughts and provide examples	
Group work to generate alternative cognitive response	
Provide clear information on behavioural responses to hot flushes	
Review relaxation and paced breathing	
Practice relaxation and paced breathing	
Elicit and respond to questions appropriately	
Outline and encourage homework and give out homework sheets and questionnaires for the next week	
Adopt empathic, positive and supportive therapeutic style	

Additional Comments:

Signed: Date:

Quality Assurance for Group CBT intervention

Independent Session Assessment
Session Four MENOS 1

Rater …………………….   Group therapist…….

Date of group………………….

**Session aims**	**Comments**
Review homework on precipitants and wellbeing goals	
Group feedback regarding cognitive work with any difficulties raised and discussed	
Practice paced breathing as a group	
Group feedback on sleep diaries	
Provide clear information about physiological aspects of sleep	
Provide clear information about factors influencing perception of sleep	
Provide information on CBT model of sleep quality and behavioural interventions	
Provide clear information about daytime tiredness	
Group discussion to generate sleep goals	
Elicit and respond to questions appropriately	
Outline and encourage homework and give out homework sheets and questionnaires for the next week	
Adopt empathic, positive and supportive therapeutic style	

Additional Comments:

Signed: Date:

Quality Assurance for Group CBT intervention

Independent Session Assessment
Session Five MENOS 1

Rater …………………….   Group therapist…….

Date of group………………….

Session aims	Comments
Review homework and progress so far to include cognitive behavioural work for hot flushes and homework from last week (sleep)	
Review paced breathing and address any difficulties	
Practice paced breathing as a group	
Provide clear information about cognitive factors affecting sleep	
Provide clear information about ways to address cognitive factors influencing sleep	
Provide clear information about factors influencing perception of sleep	
Provide clear information about cognitive behavioural strategies to address worries that cause wakefulness	
Provide clear information about managing night sweats	
Group discussion to develop sleep goals further	
Elicit and respond to questions appropriately	
Outline and encourage homework and give out homework sheets and questionnaires for the next week	
Adopt empathic, positive and supportive therapeutic style	

Additional Comments:

Signed: Date:

Quality Assurance for Group CBT intervention

Independent Session Assessment
Session Six MENOS 1

Rater ……………………. Group therapist…….

Date of group………………….

**Session aims**	**Comments**
Review homework from last week to include cognitive and behavioural sleep strategies and NS management	
Revisit cognitive behavioural model of HF/NS and link to strategies from each session	
Provide clear rationale for maintenance plan in relation to cognitive behavioural work and relapse prevention	
Deliver information around important considerations for maintenance plan	
Group to identify and discuss personal cognitive, behavioural, affective and physical signs of positive change since beginning of intervention	
Group to identify personal barriers to maintaining positive changes that may arise in future	
Group to identify personal cognitive behavioural, affective and physical signs indicative of a setback	
Group to consider ways to overcome a setback using strategies learned during the intervention	
Group members to generate and feedback personal maintenance plan using answers generated from discussion	
Elicit and respond to questions appropriately	
Practice paced breathing as a group	
Adopt empathic, positive and supportive therapeutic style	

Additional Comments:

Signed: Date:
